# Developing a Sleep Algxorithm to Support a Digital Medicine System: Noninterventional, Observational Sleep Study

**DOI:** 10.2196/62959

**Published:** 2024-12-20

**Authors:** Jeffrey M Cochran

**Affiliations:** 1 Otsuka Pharmaceutical Development & Commercialization, Inc, 508 Carnegie Center Drive, Princeton, NJ, 08540, United States, 1 609 535 9035

**Keywords:** actigraphy, machine learning, accelerometer, sleep-wake cycles, sleep monitoring, sleep quality, sleep disorder, polysomnography, wearable sensor, electrocardiogram

## Abstract

**Background:**

Sleep-wake patterns are important behavioral biomarkers for patients with serious mental illness (SMI), providing insight into their well-being. The gold standard for monitoring sleep is polysomnography (PSG), which requires a sleep lab facility; however, advances in wearable sensor technology allow for real-world sleep-wake monitoring.

**Objective:**

The goal of this study was to develop a PSG-validated sleep algorithm using accelerometer (ACC) and electrocardiogram (ECG) data from a wearable patch to accurately quantify sleep in a real-world setting.

**Methods:**

In this noninterventional, nonsignificant-risk, abbreviated investigational device exemption, single-site study, participants wore the reusable wearable sensor version 2 (RW2) patch. The RW2 patch is part of a digital medicine system (aripiprazole with sensor) designed to provide objective records of medication ingestion for patients with schizophrenia, bipolar I disorder, and major depressive disorder. This study developed a sleep algorithm from patch data and did not contain any study-related or digitized medication. Patch-acquired ACC and ECG data were compared against PSG data to build machine learning classification models to distinguish periods of wake from sleep. The PSG data provided sleep stage classifications at 30-second intervals, which were combined into 5-minute windows and labeled as sleep or wake based on the majority of sleep stages within the window. ACC and ECG features were derived for each 5-minute window. The algorithm that most accurately predicted sleep parameters against PSG data was compared to commercially available wearable devices to further benchmark model performance.

**Results:**

Of 80 participants enrolled, 60 had at least 1 night of analyzable ACC and ECG data (25 healthy volunteers and 35 participants with diagnosed SMI). Overall, 10,574 valid 5-minute windows were identified (5854 from participants with SMI), and 84% (n=8830) were classified as greater than half sleep. Of the 3 models tested, the conditional random field algorithm provided the most robust sleep-wake classification. Performance was comparable to the middle 50% of commercial devices evaluated in a recent publication, providing a sleep detection performance of 0.93 (sensitivity) and wake detection performance of 0.60 (specificity) at a prediction probability threshold of 0.75. The conditional random field algorithm retained this performance for individual sleep parameters, including total sleep time, sleep efficiency, and wake after sleep onset (within the middle 50% to top 25% of the assessed devices). The only parameter where the model performance was lower was sleep onset latency (within the bottom 25% of all comparator devices).

**Conclusions:**

Using industry-best practices, we developed a sleep algorithm for use with the RW2 patch that can accurately detect sleep and wake windows compared to PSG-labeled sleep data. This algorithm may be used for a more complete understanding of well-being for patients with SMI in a real-world setting, without the need for PSG and a sleep lab.

## Introduction

Sleep is an important behavioral biomarker for patients with serious mental illness (SMI) [1-8]. Several studies have outlined well-categorized disturbances in sleep parameters for patients with SMI [3-5,7,8]. The types of disturbances differ by condition, but many SMIs influence commonly tested sleep parameters and characteristics, including total sleep time (TST), sleep onset latency (SOL), sleep efficiency (Eff), and wake after sleep onset (WASO) [1,2,5,6,9-18].

Historically, sleep parameters were assessed primarily through sleep diaries and polysomnography (PSG); however, each of these has limitations for daily utilization [1,2]. Sleep diaries may be helpful at identifying changes in daily sleep habits and activity patterns, but as most studies highlight, these reports rely on subjective responses [1,2,5,11]. PSG requires instrumentation that may be better utilized within a dedicated sleep facility and is not designed for continuous monitoring [1,2]. Therefore, PSG findings do not fully reflect patients’ natural sleep patterns and habits.

Recent studies have focused on the role of actigraphy or the measure of relative activity, namely in sleep and wake cycles, as a means of capturing more natural sleep and wake habits [1,2,5,10-21]. This is typically accomplished utilizing accelerometer (ACC)-based data from small, portable (wristwatch-sized) recording devices. Most studies report strong to relatively strong correlation with the gold standard PSG recordings, but with the added benefit of continuous, noninvasive monitoring [1,2,10-13,15-17,19,20].

The results of studies for patients with SMI and other sleep-related conditions emphasize the value of actigraphy as a tool to gain a more complete picture of the patient’s daily health status and potentially provide insight into changes in symptomology [2,5,10,16,20,21]. However, it is important to highlight that PSG and actigraphy collect different types of data. PSG typically includes 3 separate dimensions (electrocardiography (ECG), electroencephalography, and electrooculography). Actigraphy typically provides a single dimension of ACC data (though some devices may also record heart rate data via ECG or optical modalities) that must be interpreted and processed through mathematical modeling [1,2,15-18]. Given that these data require complex postprocessing for accurate and meaningful interpretation, there is a need for the development of models that can reliably predict sleep parameters and sleep and wake windows [15,16,18,19]. This is particularly important for patients with SMI, as baseline disrupted sleep patterns often complicate accurate calculations [1,2,5,9,10,19].

Several recent studies have focused on the development of such models [10,11,15-23]; however, not all have been designed with patients who have SMI, and many have been tested only with healthy control participants. There is an additional need for models that are validated within the appropriate context of use—in this case, for patients with SMI [24].

The goal of this study was to develop a model using ACC and ECG data to accurately quantify sleep in a real-world setting. The ACC and ECG data were used to build machine learning classification models to distinguish periods of wake from periods of sleep.

## Methods

### Objectives

This was a noninterventional, nonsignificant-risk, abbreviated investigational device exemption, single-site study. The primary objective of this study was to develop a PSG-validated sleep algorithm for measuring hours of sleep at night using data from the reusable wearable sensor version 2 (RW2) patch. The RW2 patch is part of a digital medicine system (aripiprazole tablets with sensor) designed to provide objective records of medication ingestion for patients with schizophrenia, bipolar I disorder, and major depressive disorder. This study focused exclusively on the development of a sleep algorithm from the data generated by the RW2 patch and did not contain any study-related or digitized medication.

The RW2 device was manufactured by Otsuka America Pharmaceutical, Inc. (Rockville, MD, USA) and consists of an adhesive strip and a replaceable pod that collected and stored the data. This patch was placed on either side of the torso within a defined zone just above the lower edge of the rib cage.

Due to the inability to define a priori the discrepancies in model performance accuracy based on patch-derived data, this study was not specifically powered for any statistical analysis. The intent of this design was to permit ready combination with datasets of subsequent samples and sleep studies; the data generated from this study thus could be utilized to determine more precisely the required sample size for statistical analysis based on any observed limitations of the developed model.

### Participants and Recruitment

Data were collected for a total of 220 nights from 60 participants in the United States. All participants were recruited on an outpatient basis or from regional volunteers at local clinical sites in California. There was no target age range for the population, as this study was not designed as a specific intervention. Adult participants who were in good health or had a diagnosis of SMI (schizophrenia, bipolar I disorder, or major depressive disorder), as defined by the *Diagnostic and Statistical Manual of Mental Disorders, Fifth Edition*, were included in this study. Participants were excluded if they had a history of epilepsy or seizures, heart failure, or documented history of sleep apnea. Participants deemed as high risk for sleep apnea at baseline were considered on a case-by-case basis. Regular drug screens were performed to identify any substances that could impact the measurement of sleep as determined by the investigator or sponsor. Of the total participant population, 35 participants had a diagnosed SMI (12 with bipolar I disorder, 13 with schizophrenia, and 10 with major depressive disorder), and 25 participants were healthy volunteers with no SMI diagnosis.

For the literature comparison, the population (which was fully independent of that in the present study) consisted of 34 healthy adults, with mean age of 28.1 (SD 3.9) years. This population differed from that of the present study in that only healthy participants were included. Participant exclusion in the comparator study was based on a self-report questionnaire of medical history, and exclusion included history of mental health or sleep disorders [19].

### Ethical Considerations

This study was conducted in compliance with Good Clinical Practice guidelines for conducting, recording, and reporting studies, as well as for archiving essential documents. Consistent with ethical principles for the protection of human research participants, no study procedures were performed on study candidates until their written consent had been obtained. The informed consent form, protocol, and amendments for this study were submitted to and approved by an appropriate institutional review board (Aspire IRB, Santee, CA, USA), with the protocol number 031-201-00266. All participants provided written informed consent forms at the beginning of the study.

### Study Design and Treatment

This study was conducted over 6 weeks and included a 1-week observation period. The study consisted of a screening/baseline period, a 1-week observation period, and a safety follow-up call approximately 30 days after the last overnight PSG assessment, with a gap of up to 1 week between screening and the first visit to the sleep lab facility. The overall study period began with the signing of informed consent on June 28, 2019, and was completed with the last study observation on March 3, 2020. During the study, participants were allowed to take their usual medications, provided those medications were not deemed by the investigators to potentially interfere with the study measures.

In the setting of a sleep lab, participants wore the RW2 patch set in engineering mode, which enabled denser data collection than the real-world patch. The patch provided ACC and ECG data, which were used for algorithm development. PSG data were also collected as the gold standard for sleep stage classification. Participants were provided a mobile device that was paired with the wearable patch onto which the ACC and ECG data were downloaded. This device remained within Bluetooth range of the participant at all times in the sleep lab to allow continuous communication with the patch. The patch collected raw ACC and ECG data for 14 seconds, followed by being off for 6 seconds, with data being transferred from the patch to the mobile device during collection via Bluetooth. There were no memory limitations on the mobile device. Bluetooth transmission sampling rate exceeded that of the data collection rate, thus preventing data backlog on the patch.

Each participant underwent an overnight sleep assessment conducted in the sleep lab facility up to 4 times within the duration of the study. The first 3 assessment nights were followed by 1 nonassessment night at the participant’s residence, where they continued to wear the patch and maintained a sleep journal/diary (which included time of sleep and waking).

### Data Collection

#### PSG Data

**Figure 1.**
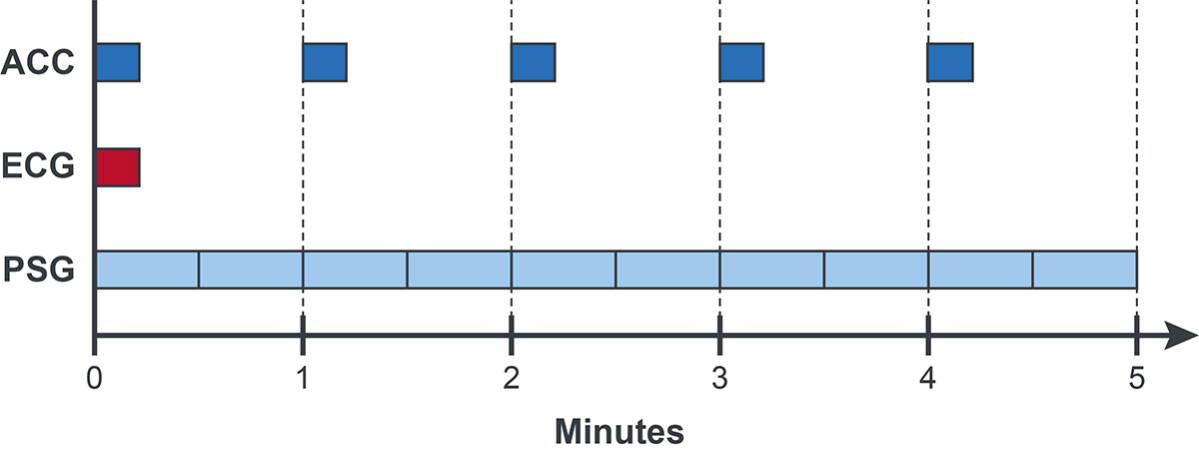
Sleep state designation per ACC, ECG, and PSG relative sampling windows. Data are displayed as aligned for ease of visualization. In early testing, data were randomly selected from 3 possible blocks per minute of ACC data and 1 block per minute of ECG data and overlaid with the corresponding PSG data to determine model sensitivity to real-world sampling. For the final algorithm, the first ACC block within each minute and the first ECG block within each 5-minute window were aligned and overlaid with the PSG data to keep the time between measurements approximately constant. ACC: accelerometer; ECG: electrocardiogram; PSG: polysomnography.

The scored PSG provided one of the following sleep stage designations at 30-second intervals: wake, non-rapid eye movement (REM) 1 sleep, non-REM 2 sleep, non-REM 3 sleep, and REM sleep. These PSG designations were then grouped to provide a binary classification at 30-second intervals: wake (wake) and sleep (non-REM 1, non-REM 2, non-REM 3, and REM). Binary classifiers were assigned for 5-minute windows of aggregated PSG data. Windows were labeled as wake if at least five of the ten 30-second PSG states in the 5-minute window were measured as wake and as sleep otherwise (Figure 1 provides more details).

#### ACC Data

Figure 1 shows that ACC data were sampled over 14 seconds within every 20-second interval, which provided three 14-second blocks of ACC data per minute. To replicate the real-world data sampling conditions (based on the sampling rate of the commercially available RW2) and determine the sensitivity of our model system to this sampling, the first 14-second ACC block within each minute was selected and all other ACC blocks within that minute were discarded. For each block, the step count, body angle, and mean X, Y, and Z acceleration features were derived. ACC features were then associated with the PSG data by aggregating the 14-second-block features over the corresponding 5-minute intervals.

#### ECG Data

ECG data were sampled over 14 seconds within every 20-second interval, providing three 14-second blocks of ECG signal data per minute. To replicate the real-world ECG data sampling paradigm and test the sensitivity of our model system to this sampling, one 14-second block from each 5-minute window was randomly selected (see Figure 1 for more details). For the final model algorithm, the first ECG block was utilized similarly to that described for the ACC data sampling above. A peak detection algorithm was applied to the signal data to identify heartbeat peaks (ie, the QRS complex within the ECG signal). The vectors of time between peaks (R-R vector) were then used to derive mean heart rate and a mean heart rate z-score (normalized to each participant’s heart rate mean and SD) for each 5-minute window.

For both the ACC and ECG data, the 5-minute window was chosen based on the sampling frequency of the real-world product, for which the patch only provides ACC data at 1-minute intervals and ECG data at 5-minute intervals. While the resolution was increased (more frequent sampling) for this study, the interval was identified as most appropriate for the collection of sufficient ACC and ECG data in the real-world application.

### Statistical Methods

Briefly, the validity of each raw waveform of the ACC and ECG data was first assessed prior to feature extraction. Raw waveforms were preprocessed with high-pass, low-pass, and notch filters, and an algorithm that had been independently validated (Otsuka Pharmaceutical Development & Commercialization, Inc.) was used to identify ECG R-peaks from the QRS complex to determine if data in the 14-second ECG data blocks were valid. The quality of patch-to-skin contact was measured through an impedance sensor on the patch. Data transformation techniques included scaling, handling of skewed data, and bias mitigation. Quality criteria were implemented to ensure calculations were not performed on overly noisy signal data, which included flags for the algorithm’s ability to distinguish heartbeat peaks from background and exclusion of blocks that resulted in physiologically unlikely heart rates (ie, <30 bpm or >200 bpm). A night was considered usable if there was at least 1 hour of valid, overlapping ACC, ECG, and PSG data.

**Figure 2.**
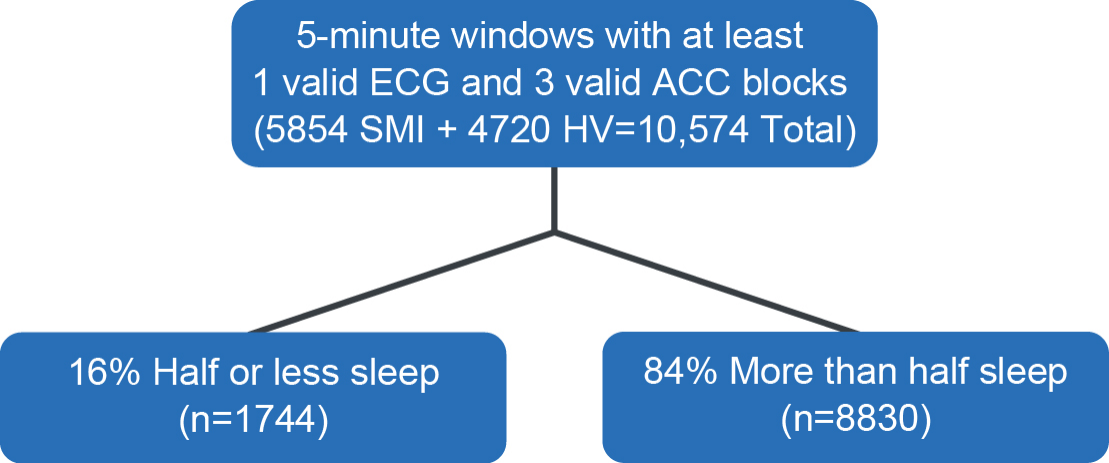
Data classification for sleep and wake window designation. ACC: accelerometer; ECG: electrocardiogram; HV: healthy volunteer; SMI: serious mental illness.

Within a 5-minute window, at least 3 blocks of ACC data (up to 5 total blocks) and at least 1 block of ECG data had to be present in order for the window to be counted as valid. Figure 2 illustrates how each block within a valid window was then classified as half-or-less sleep or more-than-half sleep. The total step count was calculated as the sum of the step counts across all ACC blocks within the 5-minute interval. For all other features, calculations were made blockwise, and the mean, SD, and range of these blockwise values were calculated over the entire 5-minute interval.

Participants were partitioned randomly into training (70%) and testing (30%) sets.

The optimal feature set was identified using a backwards propagation, greedy feature selection algorithm (based on the *F*_1_-score) on the training set only, in order to keep the test set completely independent. Feature selection methodology followed the standard procedure outlined in [25]. A light gradient boosting algorithm was trained for all 64 features, which excluded a single feature in each respective run. The model with the highest *F*_1_-score (overall model accuracy as determined by the model ability to predict and correctly assign a target state) of the 64 models that resulted was retained, and the excluded feature was removed from the feature list. This algorithm was then repeated on this model, eliminating a single feature from the remaining set each time until the optimal model performance was achieved.

Difference features (ie, the difference of features in 1 window from the corresponding features in each of the previous 5 windows) were also calculated for the selected feature subset and used as inputs to a series of classification models. If a window was preceded by a missing window (which could occur either due to missingness or to a window occurring at the beginning of a night), the difference features for the prior windows that had missing data were set to 0 (ie, they were considered to have the same value as the current window for the given feature). The final model included 13 unique features, with 5 difference features for each. These unique features are listed in Multimedia Appendix 1.

**Figure 3.**
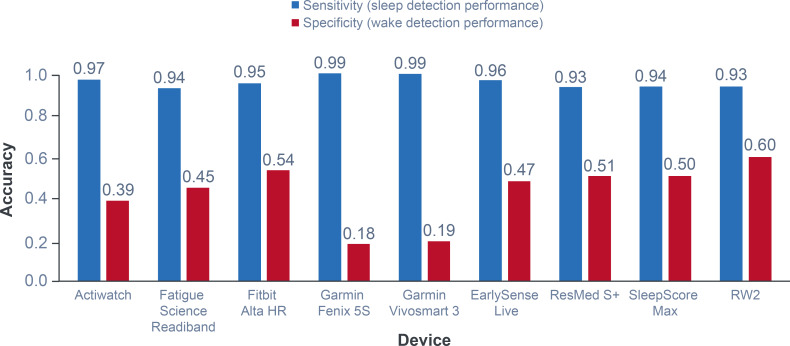
Sensitivity and specificity of the RW2 patch and literature-reported commercial comparator models, comparators from Chinoy et al [19]. Parameters were determined from the test set (n=18 participants; n=3319 analyzable windows). RW2: reusable wearable sensor version 2.

Sleep-parameter classification was performed with 3 different algorithms: light gradient boosting machine, conditional random field (CRF), and long short-term memory network. These models were chosen as each represents a highly efficient machine-learning approach to classifying sleep/wake states using a range of features; briefly, the light gradient boosting machine determines and retains the most useful features for deciding a state; CRF determines the state of a given point based on context (surrounding data); and long short-term memory determines a state based on sequential time-dependent data input [26-28]. Details on model types can be found in reviews by Sarker [26] and Woodman and Mangoni [27]. Patch-derived model performance was assessed via sensitivity and specificity metrics, and model-derived sleep parameters were compared against the corresponding PSG-derived sleep parameters (see Table 1 for more details). The most robust algorithm was further compared against 8 commercially available sleep devices reported in the literature [19] to ensure that the algorithm provided comparable performance to currently available commercial devices, which were also tested against PSG as the gold standard (see Figure 3 for more details) [19]. For this primary comparison, the individual sleep parameters were determined from a single run with the optimized algorithm in order to maintain complete independence of the test set. To further assess the robustness of the model performance, ranges and uncertainty measures for the algorithm performance were generated by re-randomizing the training and test sets in a 10-fold cross-validation analysis.

All statistical tests were computed using the Python Pingouin software package (version 0.5.3). All statistical tests for sleep parameters were chosen to match the tests chosen in the comparator publication [19].

**Table 1.** RW2^a^ patch–derived parameters reported for CRF^b^ model.

Parameter	Definition
Sensitivity	The fraction of sleep windows accurately labeled as sleep
Specificity	The fraction of wake windows accurately labeled as wake
Eff^c^	The fraction of measured time spent sleeping
SOL^d^	The time (in minutes) from the start of measurement until the participant fell asleep
WASO^e^	The time after onset of sleep that a participant spent awake
TST^f^	The total time (interrupted or uninterrupted) that a participant spent asleep

a RW2: reusable wearable sensor version 2.

b CRF: conditional random field.

c Eff: sleep efficiency.

d SOL: sleep onset latency.

e WASO: wake after sleep onset.

f TST: total sleep time.

## Results

### Participant Disposition

Of the 103 participants screened for this study, 80 were enrolled: 47 participants with SMI (16 with bipolar I disorder, 16 with schizophrenia, and 15 with major depressive disorder) and 33 healthy controls. Half (40/80, 50%) of the participants were female and male, respectively. Nearly half of all enrolled participants were Black or African American (37/80, 46.3%), and most were non-Hispanic or Latino (66/80, 82.5%). The mean age of enrolled participants was 42 years (range 20‐69 y). In total, 79 participants had at least 1 night of PSG data, and 60 of those participants (35 with SMI [12 with bipolar I disorder, 13 with schizophrenia, and 10 with major depressive disorder], 25 healthy controls) had at least 1 night of usable ACC and ECG data and were thus included in the algorithm development.

### Identification of Sleep and Wake Windows

From all ECG and ACC data samples, 73% of ECG and 98% of ACC blocks were considered usable, and, overall, for participant nights with sufficient recorded data (≥1 h of valid overlapping PSG, ECG, and ACC data), 76% of 5-minute windows were deemed analyzable. The primary reason for data exclusion in all cases was a low ECG signal-to-noise ratio resulting from poor patch contact with the skin. This resulted in the identification of 10,574 5-minute windows with at least 3 valid ACC blocks and 1 valid ECG block. Over half of these windows (n=5854, 55%) were identified in participants with SMI. Figure 2 illustrates that of these windows, 16% (n=1744) were classified as having half or less of all data labeled as sleep, and 84% (n=8830) were classified as having greater than half labeled as sleep. Table 2 details the respective number of participants and associated nights and windows for the overall, training, and test sets.

**Table 2.** Sample sizes (participants and associated nights and windows) for the training, test, and overall study populations.

Parameter		Training set, n	Test set, n	Total, n
Participants		42	18	60
Nights		118	56	174
Windows		7255	3319	10,574
	Sleep windows	6207	2623	8830
	Wake windows	1048	696	1744

### Base Model Performance

#### Sensitivity and Specificity Performance

**Figure 4.**
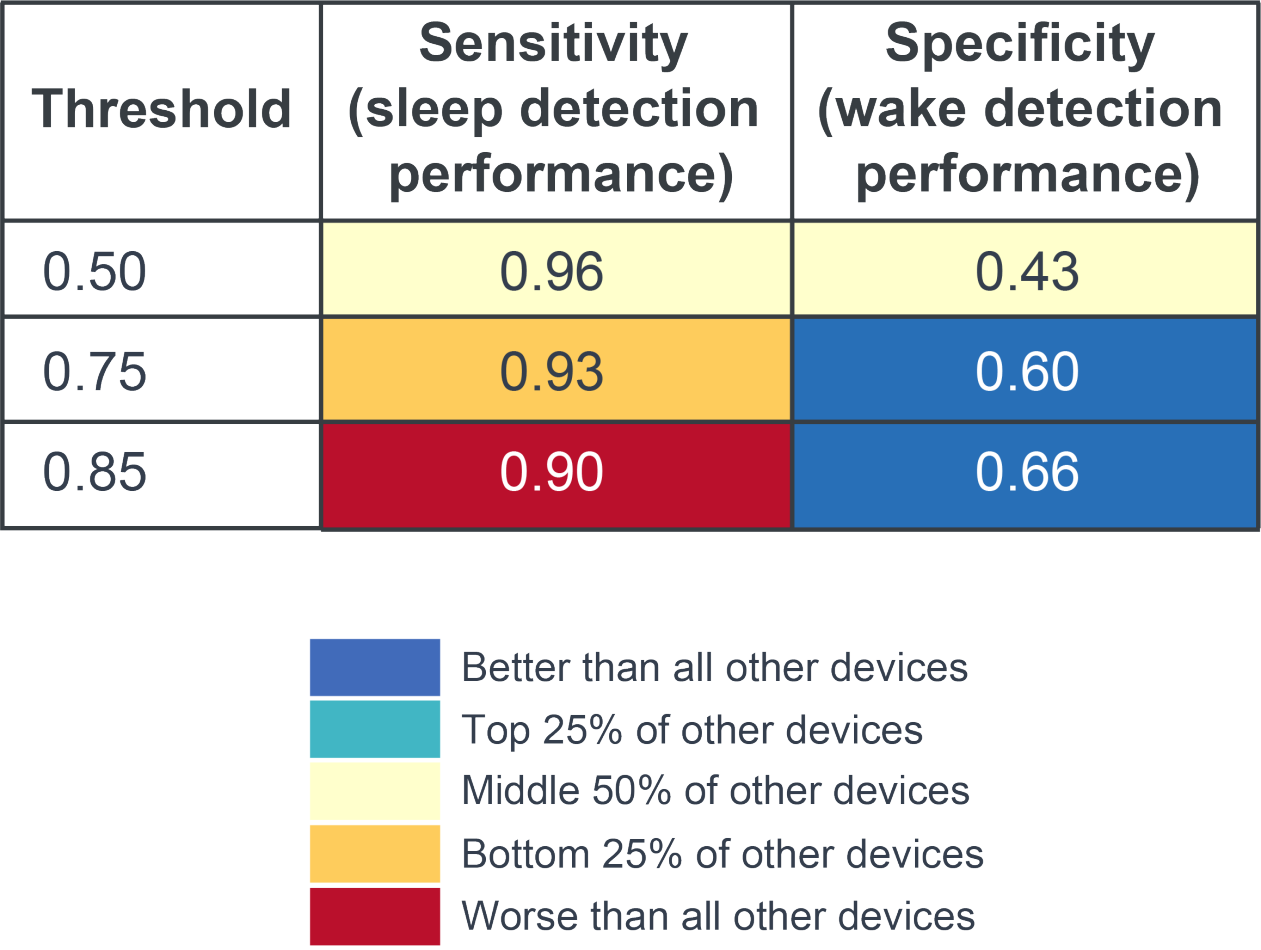
Model performance by prediction probability threshold for sensitivity (sleep detection performance) and specificity (wake detection performance); comparators from Chinoy et al [19]. Parameters were determined from the test set (n=18 participants; n=3319 analyzable windows).

Table 3 shows the area under the curve (AUC) of the overall performance metrics were similar across algorithms; however, the CRF provided a better *F*_1_-score and significantly better specificity, thus all following analyses focused on further interrogation of the CRF algorithm. Comparison of the CRF algorithm against literature reports of commercial devices indicated comparable sensitivity and specificity for a prediction probability threshold of 0.5. For thresholds above this value, wake detection performance (specificity) improved markedly, indicating improved classification accuracy for wake windows; however, this led to a decrease in accuracy for classifying sleep windows (sensitivity). Figure 4 illustrates that these findings were largely reflected in comparisons with other commercially available devices evaluated in a recent review [19].

A subgroup analysis of model performance evaluated by health status demonstrated similar AUC values for participants with SMI (AUC=0.89) and healthy participants (AUC=0.86) compared with those for the combined analysis.

**Table 3.** Model performance by algorithm type.^a^

Model	AUC^b^	*F*_1_-score^c^	Sensitivity (sleep detection performance)	Specificity (wake detection performance)
CRF^d^	0.87	0.78	0.93	0.60
LGBM^e^	0.86	0.73	0.92	0.50
LSTM^f^	0.85	0.73	0.94	0.44

a Sensitivity and specificity metrics here were calculated at a model threshold of 0.75, which maximized *F*_1_-score; parameters were determined from the test set (n=18 participants; n=3319 analyzable windows).

b AUC: area under the curve

c *F*_1_-score: harmonic mean of precision and recall.

d CRF: conditional random field.

e LGBM: light gradient boosting machine.

f LSTM: long short-term memory.

#### Sleep Parameters

**Figure 5.**
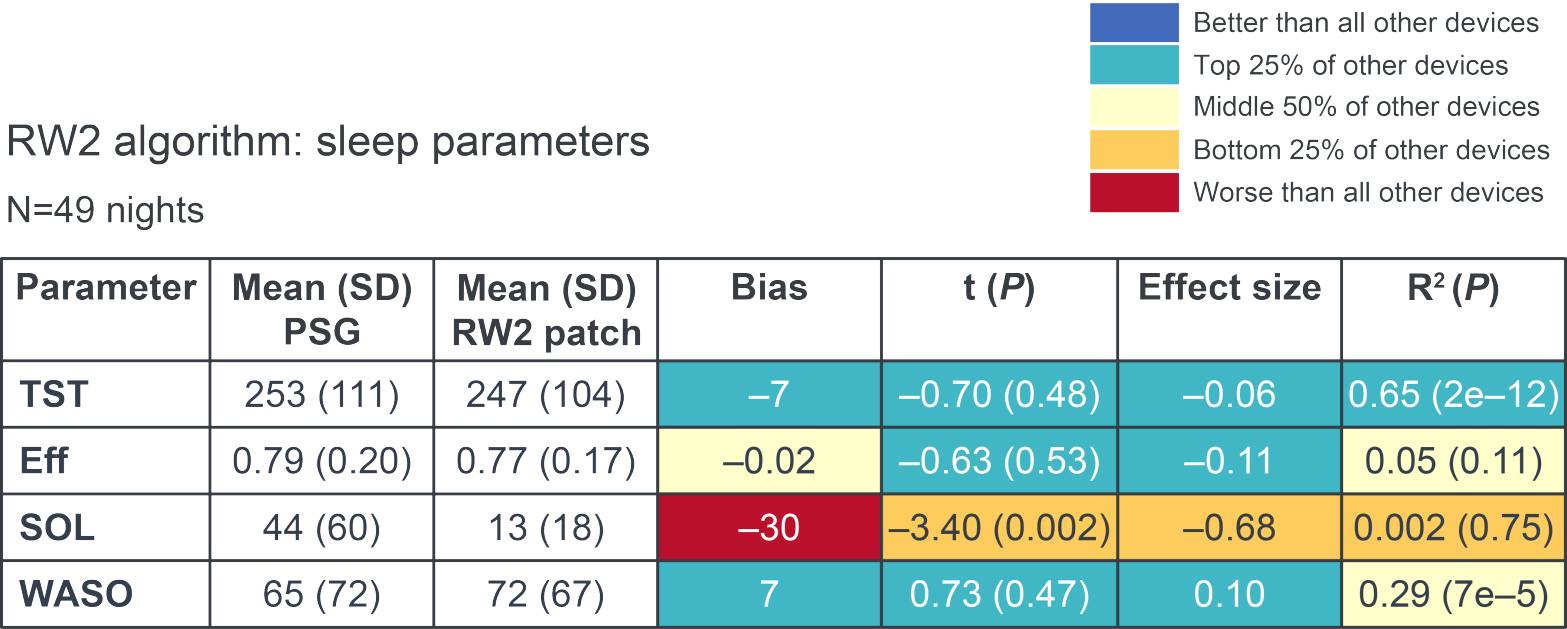
RW2 CRF algorithm performance by sleep parameters compared with PSG and commercial devices; using the Python Pingouin software package, the 2-tailed *t* test is the paired student’s *t* test (pingouin.ttest [paired=True]), the effect size is the Hedge’s *g* effect size (pingouin.compute_effsize [paired=True, eftype=“hedges”]), and the *R*^2^ is a linear regression *R*^2^ value (pingouin.linear_regression); tests were selected to directly match those chosen by the comparator publication; comparators from Chinoy et al [19]. Parameters were determined from the test set (n=18 participants; n=3319 analyzable windows). CRF: conditional random field; Eff: sleep efficiency; PSG: polysomnography; *R*^2^: proportional bias; RW2: reusable wearable sensor version 2; SOL: sleep onset latency; TST: total sleep time; WASO: wake after sleep onset.

Figure 5 shows that, for most sleep parameters (TST, Eff, and WASO) as well as fraction of time spent waking, the CRF algorithm was comparable to that of commercial devices [19]. However, the study sleep model did not perform as well as commercial devices for SOL [19].

With regard to bias and associated statistical significance, *t*(*P*), effect size, and proportional bias (*R*^2^), performance was comparable to commercial devices and in relative agreement with PSG data. Generally, the RW2 sleep model underestimated TST, Eff, and SOL, and overestimated WASO. For most parameters, calculated with a prediction probability threshold of 0.75, the relative differences between the RW2 and PSG data were minimal, except for SOL, where the RW2 model significantly underestimated latency compared with the PSG. Figure 5 displays that the sleep model fell within the top 25% of comparators for TST in all categories, for WASO in all but *R*^2^, and for Eff in *t*(*P*) and effect size. For categories in which these parameters were not within the top 25%, model performance was within the middle 50%. For SOL, the sleep model performed within the bottom 25% for *t*(*P*), effect size, and *R*^2^, and worse than all comparators for numeric bias against the PSG standard. If the RW2 algorithm instead used a prediction probability threshold of 0.5, the sleep parameters were still comparable to those reported for commercial devices, but with slightly lower performance for TST, WASO, and Eff parameters compared to the model with a prediction probability threshold of 0.75.

A 10-fold cross-validation provided similar AUC (mean 0.92, SD 0.02; IQR 0.90‐0.93), sensitivity (mean 0.95, SD 0.02; IQR 0.94‐0.96), and specificity (mean 0.58, SD 0.07; IQR 0.54‐0.62) to those observed for the single-run, fully independent test set. The full range of sleep parameters from the 10-fold cross-validation are included in Multimedia Appendix 1.

## Discussion

### Principal Results

In this study, we developed a sleep model algorithm that, when compared with PSG data, could accurately assess sleep and wake windows along with a number of common sleep parameters using ECG and ACC data collected through the RW2 (aripiprazole tablets with sensor) wearable patch. This model was developed following industry best practices, utilizing independent sets of participants for model training and testing. The model was designed for use in all participants with and without SMI, with no parametric adjustments required if participants had a diagnosis of SMI. Performance was consistent for all participants, regardless of SMI status. This validation of algorithm performance within the patient population of intended use is a key step in the development of digital measures and algorithms [24].

### Comparison to Commercial Devices and Prior Work

Compared with other commercial devices evaluated in a recent publication, the current study model had slightly worse overall accuracy but similar or better ability to detect and predict wake windows (specificity) [19]. This finding is important because the data were generally biased toward sleep data, making wake detection more difficult. The model performance was particularly strong regarding TST predictions, with similar or better performance than commercial comparators in all tested categories [19]. Similar performance was achieved for calculations of Eff and WASO, with strong performance in all tested categories, and model robustness is further supported by the consistency of results in the 10-fold cross-validation. These findings are aligned with prior studies comparing PSG and actigraphy-derived data [1,2,9,11-20]. The improved performance for classifying waking windows is promising because wake detection historically has been a weakness of actigraphy-derived sleep parameter predictions [1,10,19,20].

The model developed in this study had weak performance versus commercial comparators for SOL. This may be due to shorter TSTs for participants, compared with those reported for commercial devices used as comparators, because the data for these products exclusively reported outcomes in healthy participants [19]. Shorter sleep times may be due to baseline disrupted sleep in participants with SMI [1,2,9,10], as well as greater overall activity and shorter sleep time allotted in the sleep laboratory facility utilized in this study. Notably, sleep classification performance (ie, *F*_1_-score, sensitivity, and specificity) did not significantly differ for healthy participants and those with SMI. Potential disruptions in sleep and the observation of truncated TST mostly influenced the prediction of sleep parameters, particularly SOL. A comparison of only healthy participants with the literature was not performed for this study due to the small size of the test set. Based on the above observations, however, the test parameters would not be expected to change markedly.

Overall model performance (sleep window accuracy=96%, wake window accuracy of 43% for a prediction probability threshold of 0.5; sleep window accuracy=93%, wake window accuracy of 60% for a prediction probability threshold of 0.75) was comparable to commercially available ACC-based devices assessed in a recent review [19].

### Effect of Data Sampling Density

The study model was produced with the density of ACC and ECG data that are currently available on the RW2 patch. In real-world use of the patch, data sampling is limited to conserve battery life and extend the time a patient can wear each patch, and thus, it was important for the sleep algorithm to be sufficiently accurate using the minimum amount of available data. Ad hoc exploration of increased sampling density for ECG and ACC data did result in improvements in prediction of wake windows for increased ACC sampling, but little improvement was seen when the number of utilized ECG blocks was increased [19].

### Limitations

Recorded TSTs were shorter than those reported for other devices, most likely due to disrupted sleep patterns in participants with SMI; a majority of total sleep and wake window data were acquired from participants with SMI in this study (55%, 5854/10,574) [1,2,9,10,19]. Additionally, total sleep may have been affected by the relatively short measurement windows used in the sleep lab testing facility. This is supported by the literature; in instances where TST is lower, these parameters become more difficult to calculate [1,10,19,20]. Compounding this difficulty in calculation, poorer sleep quality led to greater uncertainties in the lower and upper limits of model bias relative to other devices, which had been tested with larger sleep windows in healthy participants without diagnosed SMI [19]. Difficulty in calculation of SOL has been previously reported for actigraphy-derived predictions of sleep parameters [1,10,19,20]. Furthermore, prior work has highlighted the difficulties associated with predictions of the most common sleep parameters for greater levels of sleep disturbance [1,10,19,20]. This may highlight a need in future work to determine features, sampling methodology, or mathematical model characteristics that can improve accuracy and reliability of sleep predictions in the face of highly disrupted or limited sleep data. However, it is important to note that sleep classification performance (ie, sensitivity, specificity, and *F*_1_-score) was not influenced by disrupted sleep patterns or truncated sleep time. Most of the primary impact of these limitations was observed in some sleep parameters, primarily SOL.

Additionally, for this investigation, the model was trained and tested on datasets from the same study; however, testing and training datasets were fully independent, following best practices.

### Conclusions

In this study, a sleep algorithm has been developed and tested for use with the RW2 ACC and ECG data that is capable of accurately detecting sleep and wake windows when compared to gold-standard PSG-derived sleep data. This algorithm followed best practices in development and testing and could be applied to all participants regardless of an SMI diagnosis. Furthermore, model performance was comparable to currently available consumer devices for sensitivity and specificity (sleep window accuracy=93%, wake window accuracy=60%), TST, Eff, and WASO.

Patients with SMI have known disrupted sleep patterns, and exacerbation of sleep disruption may indicate changes in symptomology or severity. Accurately recording sleep can provide insights into the well-being of patients with SMI and provide a more complete picture of the patient’s current health status for the care provider team. Future research should focus on sampling paradigms and modeling approaches that can accurately and reliably provide sleep parameter predictions in the face of heavily disrupted or limited sleep data.

## Supplementary material

10.2196/62959 Multimedia Appendix 1 Model feature list and associated properties, as well as 10-fold cross-validation of model sleep parameters.

## Abbreviations

ACC: accelerometer
AUC: area under the curve
CRF: conditional random field
ECG: electrocardiogram
Eff: sleep efficiency
PSG: polysomnography
*R* ^2^: proportional bias
REM: rapid eye movement
RW2: reusable wearable sensor version 2
SMI: serious mental illness
SOL: sleep onset latency
TST: total sleep time
WASO: wake after sleep onset

## References

[1] Ancoli-Israel S, Cole R, Alessi C, Chambers M, Moorcroft W, Pollak CP (2003). The role of actigraphy in the study of sleep and circadian rhythms. Sleep.

[2] Panchal P, de Queiroz Campos G, Goldman DA (2022). Toward a digital future in bipolar disorder assessment: a systematic review of disruptions in the rest-activity cycle as measured by actigraphy. Front Psychiatry.

[3] Krystal AD (2012). Psychiatric disorders and sleep. Neurol Clin.

[4] Faulkner S, Didikoglu A, Byrne R, Drake R, Bee P (2023). Light-dark and activity rhythm therapy (L-DART) to improve sleep in people with schizophrenia spectrum disorders: a single-group mixed methods study of feasibility, acceptability and adherence. Clocks Sleep.

[5] Kawai K, Iwamoto K, Miyata S (2023). Comparison of polysomnography, single-channel electroencephalogram, Fitbit, and sleep logs in patients with psychiatric disorders: cross-sectional study. J Med Internet Res.

[6] Boivin DB (2000). Influence of sleep-wake and circadian rhythm disturbances in psychiatric disorders. J Psychiatry Neurosci.

[7] Talih F, Ajaltouni J, Ghandour H, Abu-Mohammad AS, Kobeissy F (2018). Insomnia in hospitalized psychiatric patients: prevalence and associated factors. Neuropsychiatr Dis Treat.

[8] Carney CE, Edinger JD, Kuchibhatla M (2017). Cognitive behavioral insomnia therapy for those with insomnia and depression: a randomized controlled clinical trial. Sleep.

[9] Cochran JM, Heidary Z, Knights J (2021). Characterization of activity behavior using a digital medicine system and comparison to medication ingestion in patients with serious mental illness. NPJ Digit Med.

[10] Heidary Z, Cochran JM, Peters-Strickland T, Knights J (2021). A rest quality metric using a cluster-based analysis of accelerometer data and correlation with digital medicine ingestion data: algorithm development. JMIR Form Res.

[11] Kainec KA, Caccavaro J, Barnes M, Hoff C, Berlin A, Spencer RMC (2024). Evaluating accuracy in five commercial sleep-tracking devices compared to research-grade actigraphy and polysomnography. Sensors (Basel).

[12] Lee XK, Chee NIYN, Ong JL (2019). Validation of a consumer sleep wearable device with actigraphy and polysomnography in adolescents across sleep opportunity manipulations. J Clin Sleep Med.

[13] Liang Z, Chapa Martell MA (2018). Validity of consumer activity wristbands and wearable EEG for measuring overall sleep parameters and sleep structure in free-living conditions. J Healthc Inform Res.

[14] Kubala AG, Barone Gibbs B, Buysse DJ, Patel SR, Hall MH, Kline CE (2020). Field-based measurement of sleep: agreement between six commercial activity monitors and a validated accelerometer. Behav Sleep Med.

[15] Roberts DM, Schade MM, Master L (2023). Performance of an open machine learning model to classify sleep/wake from actigraphy across ∼24-hour intervals without knowledge of rest timing. Sleep Health.

[16] Banfi T, Valigi N, di Galante M, d’Ascanio P, Ciuti G, Faraguna U (2021). Efficient embedded sleep wake classification for open-source actigraphy. Sci Rep.

[17] Quante M, Kaplan ER, Cailler M (2018). Actigraphy-based sleep estimation in adolescents and adults: a comparison with polysomnography using two scoring algorithms. Nat Sci Sleep.

[18] Nakazaki K, Kitamura S, Motomura Y (2014). Validity of an algorithm for determining sleep/wake states using a new actigraph. J Physiol Anthropol.

[19] Chinoy ED, Cuellar JA, Huwa KE (2021). Performance of seven consumer sleep-tracking devices compared with polysomnography. Sleep.

[20] Hedner J, Pillar G, Pittman SD, Zou D, Grote L, White DP (2004). A novel adaptive wrist actigraphy algorithm for sleep-wake assessment in sleep apnea patients. Sleep.

[21] Yokoyama S, Kagawa F, Takamura M (2023). Day-to-day regularity and diurnal switching of physical activity reduce depression-related behaviors: a time-series analysis of wearable device data. BMC Public Health.

[22] Elsenbruch S, Harnish MJ, Orr WC (1999). Heart rate variability during waking and sleep in healthy males and females. Sleep.

[23] Geng D, An Q, Fu Z, Wang C, An H (2023). Identification of major depression patients using machine learning models based on heart rate variability during sleep stages for pre-hospital screening. Comput Biol Med.

[24] Goldsack JC, Coravos A, Bakker JP (2020). Verification, analytical validation, and clinical validation (V3): the foundation of determining fit-for-purpose for Biometric Monitoring Technologies (BioMeTs). NPJ Digit Med.

[25] Kuhn M, Johnson K (2019). Feature Engineering and Selection: A Practical Approach for Predictive Models.

[26] Sarker IH (2021). Machine learning: algorithms, real-world applications and research directions. SN Comput Sci.

[27] Woodman RJ, Mangoni AA (2023). A comprehensive review of machine learning algorithms and their application in geriatric medicine: present and future. Aging Clin Exp Res.

[28] Lafferty JD, McCallum A, Pereira FCN Conditional random fields: probabilistic models for segmenting and labeling sequence data.

[29] Otsuka Pharmaceutical Clinical Trial Data Transparency.

