# Supplementary material for: Developing a Sleep Algxorithm to Support a Digital Medicine System: Noninterventional, Observational Sleep Study
Source: JMIR Ment Health. 2024 Dec 20;11:e62959. doi: 10.2196/62959 (PMC11683743; doi:10.2196/62959)
Supplement: Multimedia Appendix 1 [file mental-v11-e62959-s001.doc]

### Multimedia Appendix 1

Table S1. Model feature list and associated properties.a

| **Variable Name** | **Feature** | **Feature Calculation Description** |
| --- | --- | --- |
| 'absAng_d', | Absolute Value of Body Angle - Range | Calculate the absolute value for each measurement of body angle in the window, identify the maximum and minimum values of these absolute values of body angles and then find the difference (maximum − minimum) |
| 'absXMean_s', | Absolute Value of Mean X-Acceleration – Standard deviation | Calculate the absolute value for each measurement of Mean X-Acceleration in the window and calculate the standard deviation of these values |
| 'yMean_d', | Mean Y-Acceleration – Range | Identify the minimum and maximum values of the Mean Y-Acceleration values in the window and calculate the difference (maximum − minimum) |
| 'angle_s', | Body Angle – Standard deviation | Calculate the standard deviation of all measurements of body angle in the window |
| 'zMean_m', | Mean Z-Acceleration – Mean | Calculate the mean of all measurements of Mean Z-Acceleration |
| 'absAng_s', | Absolute Value of Body Angle –  Standard deviation | Calculate the absolute value for each measurement of body angle in the window and calculate the standard deviation of these values |
| 'angle_d', | Body Angle – Range | Identify the minimum and maximum values of the body angle values in the window and calculate the difference (maximum − minimum) |
| 'accMean_s', | Mean Total Acceleration –  Standard deviation | Calculate the total acceleration for each acceleration measurement in the window (take the square root of ((Mean X-Acceleration)^2^ + (Mean Y-Acceleration)^2^ + (Mean Z-Acceleration)^2^) and then calculate the standard deviation of these values |
| 'ECG_Rate_Mean_z', | Mean Heart Rate Z-Score | hr_m_z = (hr - hr_mean)/(hr_std) where "hr" is the heart rate value in the current window, "hr_mean" is the mean heart rate from all prior windows for that patient, and "hr_std" is the standard deviation of the heart rate for all prior windows for that patient |
| 'yMean_s', | Mean Y-Acceleration – Standard deviation | Calculate the standard deviation of all measurements of Mean Y-Acceleration in the window |
| 'absAng_m', | Absolute Value of Body Angle - Mean | Calculate the absolute value for each measurement of body angle in the window and calculate the mean of these values |
| 'zMean_d', | Mean Z-Acceleration - Range | Identify the minimum and maximum values of the Mean Z-Acceleration values in the window and calculate the difference (maximum − minimum) |
| 'xMean_d' | Mean X-Acceleration - Range | Identify the minimum and maximum values of the Mean X-Acceleration values in the window and calculate the difference (maximum − minimum) |
| 'absAng_m_all' | Absolute Value of Body Angle - Mean | Calculate the absolute value for each measurement of body angle in the window and calculate the mean of these values - this version of the feature is not passed into the CRF function, but instead kept in its raw form for a given window and passed through to be used for post-processing of the CRF output |
| 'steps_all' | Sum of Steps | Sum the total step count of all measurements in a window - this feature is not passed into the CRF function, but instead kept in its raw form for a given window and passed through to be used for post-processing of the CRF output |

^a^All means and standard deviations were calculated using numpy.mean and numpy.std, respectively, in Python.
CRF, condition random field; d, difference; hr, heart rate; m, mean; s, standard deviation, z, Z-score.

Table S2. Ten-fold cross-validation of model sleep parameters at model threshold of 0.5.

| **Parameter** | **Mean (SD)** | **IQR** |
| --- | --- | --- |
| AUC | 0.92 (0.02) | 0.90–0.93 |
| Sensitivity | 0.95 (0.02) | 0.94–0.96 |
| Specificity | 0.58 (0.07) | 0.54–0.62 |
| Eff  Bias  T-Test Statistic  T-Test *P*-Value  Hedge’s Effect Size  Regression R^2^  Regression *P*-Value | 0.035 (0.017)  1.79 (1.08)  0.238 (0.275)  0.23 (0.12)  0.32 (0.19)  0.01 (0.04) | 0.021 – 0.047  0.86 – 2.85  0.009 – 0.41  0.13 – 0.30  0.20 – 0.48  3e-6 – 1e-3 |
| SOL  Bias  T-Test Statistic  T-Test *P*-Value  Hedge’s Effect Size  Regression R^2^  Regression *P*-Value | 7 (5)  1.32 (0.90)  0.329 (0.303)  0.17 (0.11)  0.36 (0.27)  0.16 (0.32) | 4 – 10  0.70 – 1.78  0.084 – 0.491  0.10 – 0.26  0.13 – 0.64  2e-11 – 2e-2 |
| WASO  Bias  T-Test Statistic  T-Test *P*-Value  Hedge’s Effect Size  Regression R^2^  Regression *P*-Value | 11 (5)  1.81 (1.18)  0.259 (0.302)  0.21 (0.11)  0.41 (0.19)  0.007 (0.021) | 8 – 15  0.78 – 3.08  0.004 – 0.438  0.13 – 0.29  0.26 – 0.61  2e-9 – 2e-4 |
| TST  Bias  T-Test Statistic  T-Test *P*-Value  Hedge’s Effect Size  Regression R^2^  Regression *P*-Value | 11 (5)  1.82 (1.19)  0.259 (0.302)  0.12 (0.06)  0.75 (0.14)  1e-9 (3e-9) | 8 – 15  0.78 – 3.14  0.003 – 0.437  0.08 – 0.16  0.62 – 0.89  5e-23 – 4e-11 |

AUC, area under the curve; Eff, sleep efficiency; IQR, interquartile range; SD, standard deviation; SOL, sleep onset latency; TST, total sleep time; WASO, wake after sleep onset.
